# High Quality Pt–Pt Metal Bonding for High Temperature Packaging

**DOI:** 10.3390/mi13091543

**Published:** 2022-09-17

**Authors:** Jiazheng Liu, Junqiang Wang, Mengwei Li, Haikun Zhang

**Affiliations:** 1 Academy for Advanced Interdisciplinary Research, North University of China, Taiyuan 030051, China; 2 Notional Key Laboratory of Instrumentation Science & Dynamic Measurement, North University of China, Taiyuan 030051, China

**Keywords:** Pt–Pt interconnection, high-temperature resistant packaging, metallic bonding

## Abstract

Platinum is an ideal material for high-temperature resistant device packaging due to its higher melting point and good electrical properties. In this paper, the thermocompression bonding of Pt–Pt metal electrodes was successfully realized through process exploration, and the package interconnection that meets the requirements was formed. A square bump with a side length of 160 µm and a sealing ring with a width of 80 µm were fabricated by magnetron sputtering. Different pressure parameters were selected for chip-level bonding; the bonding temperature was 350 °C for about 20 min. Analysis of the interface under a scanning electron microscope found that the metal Cr diffused into Pt. It was found that two chips sputtered with 300 nm metal Pt can achieve shear resistance up to 30 MPa by flip-chip bonding at 350 °C and 100 MPa temperature and pressure, respectively. The leakage rate of the sample is less than 2 × 10^–3^ Pa·cm^3^/s, the bonding interface is relatively smooth, and the hot-pressed metal bonding of Pt electrodes with good quality is realized. By comparing the failure rates at different temperatures and pressures, the process parameters for Pt–Pt bonding with higher success rates were obtained. We hope to provide new ideas and methods for the packaging of high-temperature resistant devices.

## 1. Introduction

Sensors working with graphene as a sensitive material have received extensive attention in recent years. The unique thermal properties, electrical properties, and high-temperature resistance [1,2,3] of graphene show a strong potential for enhancing performance and improving the reliability of devices operating at high temperatures and harsh environments. However, the lack of reliable high-temperature packaging technology impedes the application of graphene in the field of high-temperature MEMS devices. Burla et al. [4] achieved nickel wire bonding for high-temperature packaging, and Ni wire bonds were found to be electrically stable for temperatures up to 550 °C. However, the high-temperature oxidation of nickel limits its practical application. On the other hand, Pt has almost perfect corrosion resistance, making it a better high-temperature encapsulation material than nickel. Brachmann et al. [5] demonstrated a Pt wire bonding method that can withstand an 1100 °C environment. The investigated Pt films were composed of a 50 nm thick e-beam evaporated Cr seed layers and an approximately 1 µm electrodeposited Pt film. This study proved the excellent prospects of Pt in the field of high-temperature packaging. To the best of our knowledge, the current high-temperature packaging mostly uses wire bonding technology, while wire bonding technology can no longer meet the requirements of miniaturization, light weight, high performance, and low power consumption of modern electronic products [6]. Notably, the flip chip is obviously more in line with the future development trend of the electronics industry.

The common electronic packaging method is realized by forming flip chips [7,8] by thermocompression metallic bonding [9] or direct bonding [10]. The essence of metallic bonding is the mutual diffusion of atoms on the surface of two metals [9], which relies on metallic bonds, metal melting, and other factors in order to bond firmly. On the one hand, it combines the excellent properties of the material and, on the other hand, completely utilizes the benefits of the metal film to improve the photoelectric performance of the device. Direct bonding involves cleaning and activating the surface of the bonding sheet [11], directly bonding it at room temperature, and finally combining it with heat treatment to form an interconnected interface. The difference between the two methods is that metallic bonding involves the same kind of metal material, while direct bonding can be carried out with two different materials. Therefore, defects such as the dislocations generated in the process of metal bonding only exist near the interface of metal bonding and do not extend to the entire material and thus hardly affect the performance of the material before bonding. Since a thin oxide film is formed on the surface of the metal in the air, the oxide film blocks the mutual diffusion of atoms on the two metal surfaces, restricting the diffusion of atoms unconditionally. Additionally, the bonding interface of metal bonding cannot add a dielectric layer. Metal bonding is usually achieved by heating and pressing and is different from the eutectic interface interconnection formed between different metals [12]. For example, Au–Au, Cu–Cu [13,14] and Al–Al [15,16] make atomic-level contact under the simultaneous action of heat and pressure. Under the movement of atoms, the two layers of metals undergo diffusion movement, and the diffused atoms connect the two layers of metals together.

In this paper, we have focused on the high-temperature packaging needs of MEMS devices [17,18,19] and used Pt with excellent performance at high temperatures as the bonding material [20,21] to explore the process required for its packaging and its performance after packaging.

## 2. Materials and Methods

There are three steps to realize Pt–Pt interconnection: test sample design, fabrication of fine-pitch bumps followed by bumps surface pretreatment, and finally Pt–Pt thermocompression bonding.

### 2.1. Test Sample Design

**Figure 1:**
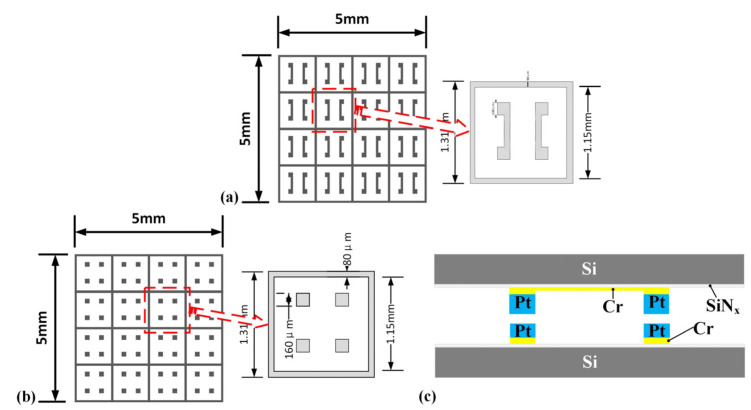
Schematic diagram of mask and bonding structure. (**a**) Mask of the top substrate. (**b**) Mask of the bottom substrate. (**c**) Cross-sectional diagram of bonded structure.

The top and low substrates of the bonding are two chips of 6 × 6 mm^2^ and 8 × 8 mm^2^, respectively. The total bonding area is approximate to 5.5 mm^2^. The schematic diagram of the mask is shown in Figure 1. The outer side length of the sealing ring of the single repeating unit is 1.31 mm, the inner side length is 1.15 mm, and the side length of the small square of the bonding bump is 160 µm. The side length of the total mask structure is 5 × 5 mm, and each structure contains 16 repeating units. The figure on the right of Figure 1a shows a single, repeating unit, which is located on a substrate with a size of 8 × 8 mm. The figure on the right of Figure 1b shows a single, repeating unit, which is located on a substrate with a size of 6 × 6 mm. The above shapes are made of silicon as the substrate through photolithography, sputtering, and other processes. The lithography was performed using an MA6 model lithography machine produced by SUSS MicroTec in Germany. The top and bottom two substrates are bonded by thermocompression to ensure a firm connection between the sealing ring and the bump, forming a closed space. 

### 2.2. Substrate Fabrication

**Figure 2:**
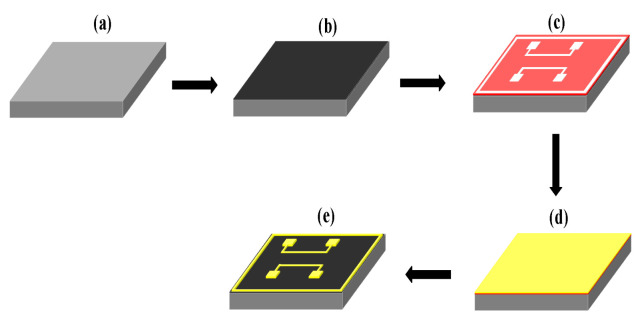
Schematic of the process for fabrication of the top substrate. (**a**) Cleaning. (**b**) SiNx passivation layer deposited by PECVD. (**c**) Negative photoresist masks and lithography. (**d**) Sputtering Cr/Pt electrodes. (**e**) Negative adhesive stripping.

The fabrication process of the top substrate is shown in Figure 2. First, the 400 µm thick silicon wafers were routinely cleaned, followed by ultrasonic cleaning with acetone, isopropanol, and water for 5 min each, and finally dried under N2 [Figure 2a] [22]. A 300 nm thick SiNx passivation layer was first deposited on a 400 µm thick Si wafer by plasma-enhanced chemical vapor deposition (PECVD), and a mask structure was created on the SiNx passivation layer using a negative photoresist. Subsequently, the bottom Cr/Pt electrodes with thicknesses of 50 and 300 nm, respectively, were deposited on the SiNx layer by magnetron sputtering [Figure 2b]. The electrode should not be too thick because an excessively thick bonding interface layer may cause the formation of microcracks and lead to poor bonding quality [23,24]. Finally, the negative adhesive peeling was completed in acetone to produce the top substrate. The process for fabrication of the bottom substrate is the same as that of the top substrate.

To remove surface oxide film, both the top substrate and bottom substrate were pretreated by Ar (a small amount of H2) plasma with a gas flow rate of 250 sccm under the power of 200 W for 180 s. After the metal surface is activated, the degree of atomic diffusion is increased by heating and pressing to tightly combine the two structures. The bonding adopts the electronic packaging FC150 flip-chip welding machine.

### 2.3. Pt-Pt Thermocompression Bonding

The relationship between bonding time and temperature is shown in Table 1. The present work attempts to use four sets of bonding parameters to determine the range of parameters that can achieve better Pt–Pt bonding interconnections. There are two purposes for using these four sets of parameters. On the one hand, it can be identified whether the interconnection interface can be formed after bonding, and on the other hand, whether the shear resistance of the sample after bonding is good within this pressure range can be ascertained. Finally, parameters that can form an interconnected interface and have a certain shear resistance are selected. The variables of these four sets of parameters are all pressure, the temperature is 350 °C, and the bonding time is 1200 s. Due to the high melting point of platinum, it is more difficult to bond by thermocompression than other metals such as gold and copper. Therefore, when flip-chip welding is used for thermocompression bonding, the bonding temperature and bonding time are both selected to be close to the upper limit of the instrument.

From Figure 1a,b, the bonding area of the bonding pair can be calculated to be about 5.5 mm^2^. According to the formula P = F/S, the pressure of case 1 is 30 MPa, the pressure of case 2 is 50 MPa, the pressure of case 3 is 80 MPa, and the pressure of case 4 is 100 MPa during thermocompression bonding.

## 3. Results and Discussion

Four different tests, including interfacial analysis, shear strength analysis, hermeticity detection, and failure analysis were performed to evaluate the bonding performance.

### 3.1. Interfacial Analysis

**Figure 3:**
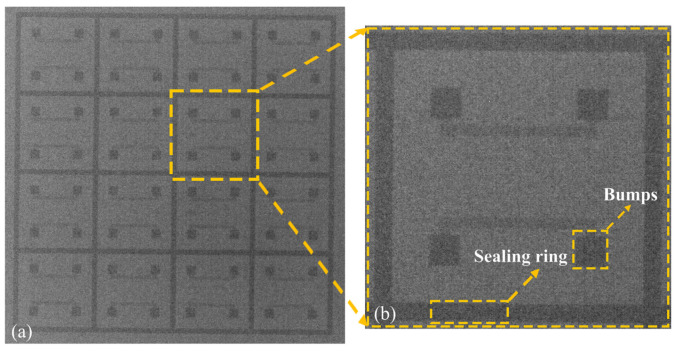
X-ray image. (**a**) Overall SEM image of the chip. (**b**) Enlarged view of a single unit.

After polishing at the 10 µm and 1 µm fine levels, the cross-sectional interface of Pt–Pt bonding under SEM can be clearly seen in Figure 3. It can be observed from the backscatter mode of the electron microscope that the bonding interface is relatively flat with no obvious cracks or gaps. This suggests that after the thermocompression bonding described in this article, the Pt–Pt metal electrodes are interconnected.

**Figure 4:**
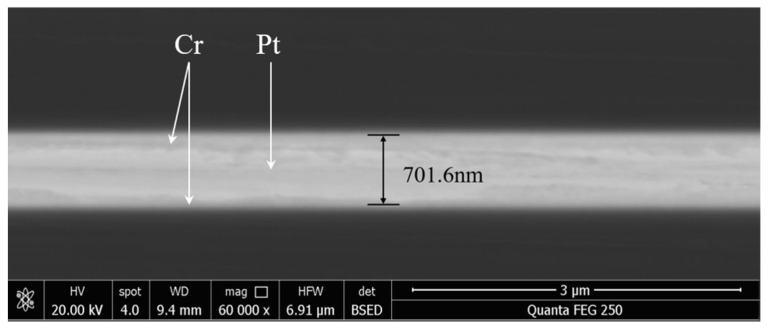
SEM image of the bump.

Figure 4 is the bonding image of the bump obtained by the SEM mode under the electronic scanning electron microscope. The bright part in the middle is the metal layer. The thickness of the metal layer is measured to be about 700 nm, which is consistent with the thickness of 50 nm Cr and 300 nm Pt plated during sputtering. Further, no obvious cracks were observed in the metal layer, and the whole surface tends to be smooth, forming a good interconnection interface. Interestingly, no obvious delamination phenomenon of Cr and Pt resulting from the diffusion of Cr is observed in the metal region on each side.

**Figure 5:**
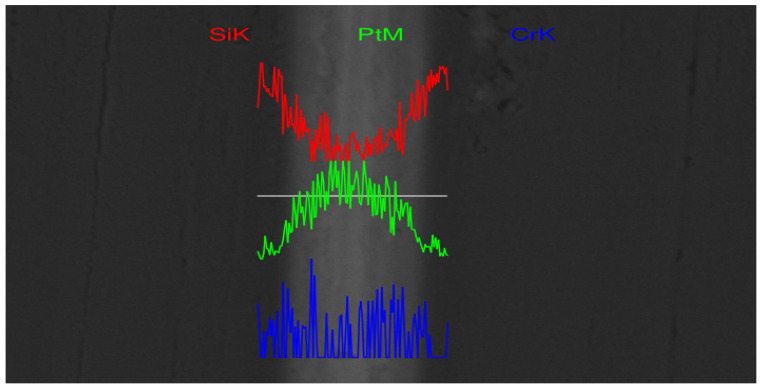
EDS line scan of the bonding interface.

Figure 5 shows the result of the line scan of the bonding interface. Through the longitudinal line scan of the bonding interface, the content of three elements is analyzed. It can be found that most of the Si elements are distributed on both sides of the metal layer, which is more in line with the actual situation, indicating that the hot-press bonding does not cause the non-metallic surface to diffuse inward. Moreover, the content of Pt element is mainly concentrated in the metal layer area, the middle part is more concentrated, and the two sides are more symmetrical and uniform, which shows that the interconnection interface formed after thermocompression bonding has no displacement, and the Pt–Pt interface is better connected with fewer impurities. Additionally, the widely distributed Cr element is essentially more concentrated between the Si layer and the Pt layer, and is consistent with the SEM image shown in Figure 4. The Cr layer is sandwiched between the metal layer and the dielectric layer as an adhesion layer. After thermocompression bonding, a small amount of diffusion of Cr element occurred between the Pt metal layers.

**Figure 6:**
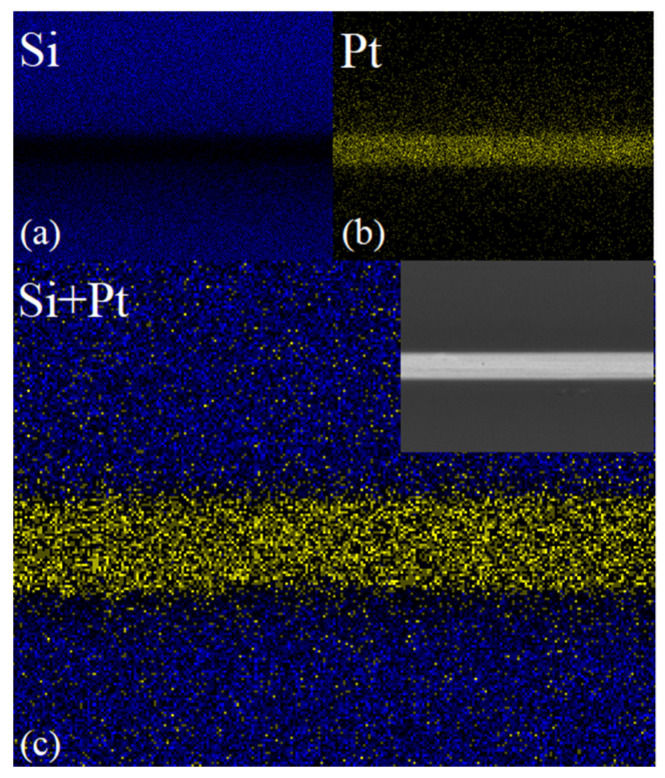
EDS surface scanning element analysis diagram of the bonding interface. (**a**) Distribution diagram of silicon element, (**b**) distribution diagram of platinum element, (**c**) consolidated diagram of element distribution.

Figure 6 is the distribution of silicon and Pt in the backscattered SEM image of the interconnect interface. The blue dots in Figure 6a represent the distribution of silicon, and the yellow dots in Figure 6b indicate the distributed Pt, and Figure 6c shows the effect of integrating the two SEM images. Through elemental surface scanning analysis, it can be observed that the bonded Pt element is still in the bonding area with no dislocation or drift.

**Figure 7:**
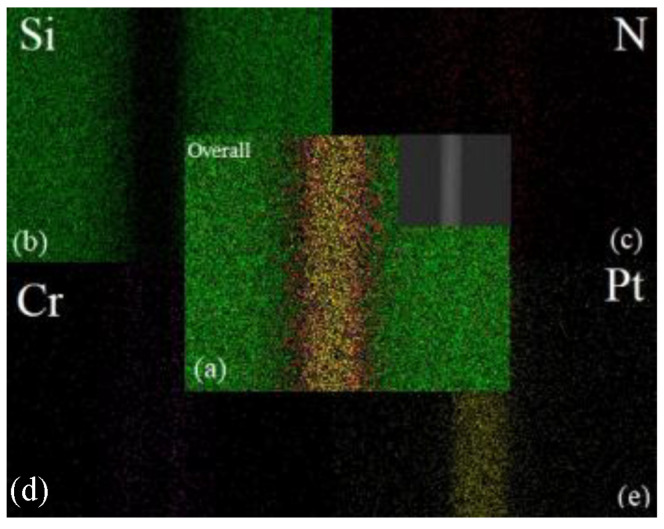
EDS surface scanning element analysis diagram of the bonding interface. (**a**) Consolidated diagram of element distribution, (**b**) distribution diagram of silicon element, (**c**) distribution diagram of nitrogen element, (**d**) distribution diagram of chromium element, (**e**) distribution diagram of platinum element.

In Figure 7a, the green dots in Figure 7b represent the Si element, the red dots in Figure 7c represent the N element, the pink dots in Figure 7d represent the Cr element, and the yellow dots in Figure 7e represent the Pt element; the yellow dots representing the Pt element, Figure 7a, are a visual SEM image of the final integration of each element. The combination of (Si + Cr + Pt = 100) and (Si + N + Cr + Pt = 100) elements were selected for analysis. The delamination of each element in the bonded sample was more obvious and no dislocation diffusion or element drift was observed.

### 3.2. Shear Strength Analysis

**Figure 8:**
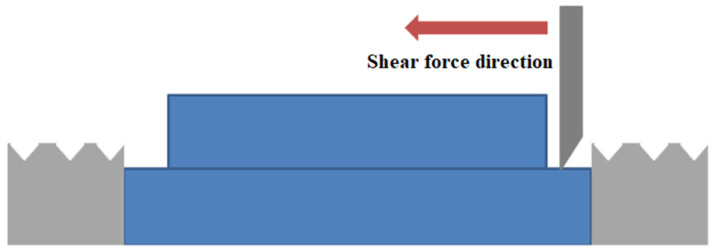
Schematic diagram of shear force test.

Figure 8 is a schematic diagram of the shear force test. The equipment used in the experiment is Dage4000 bond tester which can provide testing of bond shear force and tensile force. The sample in the picture is denoted by the blue part in the middle. After the lower substrate is fixed, the push knife moves in the horizontal direction until the upper and lower bonded substrates are separated. At the moment of separation, the force required to stop the pushing knife is the force of bonding at that moment.

**Figure 9:**
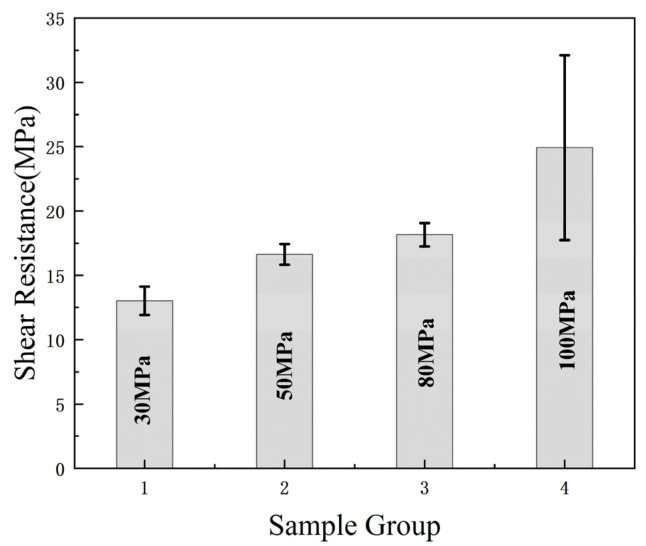
Bonding strength of substrates.

Based on theoretical inferences, increasing the pressure can enhance the bonding strength. Importantly, the pressure applied should not exceed the threshold of the substrate’s withstanding ability. Excessive pressure may cause an overflow of sputtered metal or cracks in the substrate. The shear resistance test in the effectively bonded sample in this experiment is shown in Figure 9. A total of 12 bonding samples were selected and divided into four groups according to the different bonding pressures: 30 MPa, 50 MPa, 80 MPa, and 100 MPa. Their shear resistance was found to be in the range of 12.2–14.3 MPa, 15.1–17.6 MPa, 16.9–18.9 MPa, and 17.3–31.8 MPa. The bonding pressure is positively correlated within the withstandable range of the substrate, and its shear resistance can reach a maximum of 30 Mpa as the pressure increases. When the bonding pressure is 100 MPa, the measured value is the maximum shear force that the silicon wafer can withstand, and the average shear resistance can reach 25 MPa; thereby, the shear strength of Pt–Pt bonding meets the standard.

**Figure 10:**
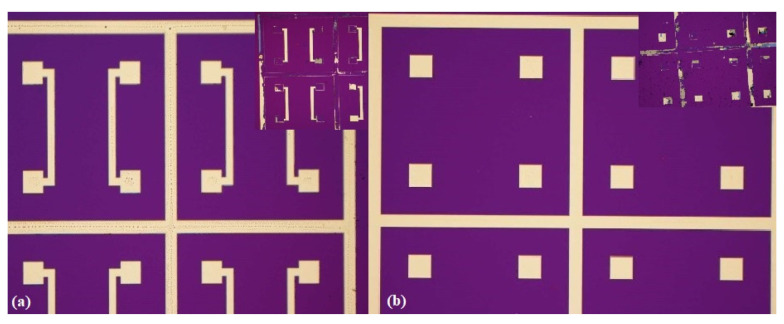
Shear force test comparison image. (**a**) Top substrate. (**b**) Bottom substrate.

Figure 10 shows the microscope schematic diagrams of the Pt metal sealing ring before thermocompression bonding and the Pt metal sealing ring of the debris after the shear force test. The shear test is generally divided into three fracture modes: IMC mode, solder mode, and mixed IMC/solder mode. Fractures in the IMC mode generally occur in the IMC layer. As can be observed from the Figure 10, the shear test destroyed the bonding electrode and the sealing ring, and it can be seen that the fracture mode in the test is mainly the IMC mode [25,26], indicating that the shear resistance is mainly due to the force exerted on the bonding electrode. This demonstrates that the bonding is effective.

Of course, some metals do not perform well in shear tests. This may be caused by uneven sputtering during the coating process due to the influence of experimental factors.

### 3.3. Hermeticity Detection

**Figure 11:**
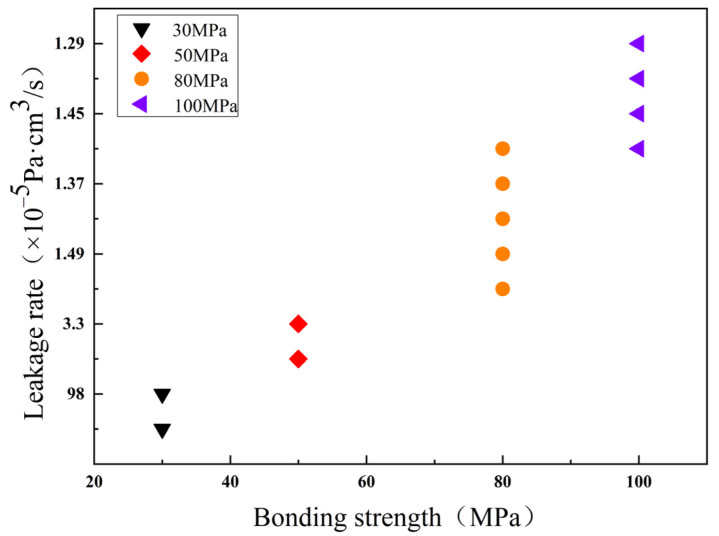
Influence of pressure on bonding leakage rate.

According to the method defined by the inspection standard (GJB 548B-2005 method 1014.2), the purpose of the test is to determine the hermeticity of microelectronic and semiconductor device packages with internal cavities. The hermeticity test of the four groups of bonded pairs is carried out. First, a detailed inspection was carried out using the ZHP-30D helium mass spectrometer leak detector. The sample is kept under pressure of 4 × 10^–5^ Pa for 2 h, and the leakage rate is measured with the leak detector after taking it out. The actual leakage rate of the technically required samples is less than 2 × 10^–3^ Pa·cm^3^/s, that is, less than the specified leakage rate value (5 × 10^–3^ Pa·cm^3^/s). As Table 2 shown, the first group of samples through the experiment showed the minimum leakage rate measured to be 30 MPa/165 N, 350 °C, 1200 s: 3.3 × 10^–4^ Pa·cm^3^/s, the maximum leakage rate: 9.8 × 10^–4^ Pa·cm^3^/s, the average leakage rate: 6.55 × 10^–4^ Pa·cm^3^/s, which is less than the leakage rate value required by the specification. Similarly, the second group of samples showed the leakage rate measured under the bonding conditions of 50 MPa/275 N, 350 °C, 1200 s: 5.9 × 10^–4^ Pa·cm^3^/s, 3.3 × 10^–5^ Pa·cm^3^/s, average leakage rate: 3.115 × 10^–4^ Pa·cm^3^/s, which is less than the specified leakage rate value. The third group of samples: 80 MPa/550 N, 350 °C, the maximum leakage rate measured under the bonding condition of 1200 s: 2.77 × 10^–5^ Pa·cm^3^/s, the minimum leakage rate: 1.37 × 10^–5^ Pa·cm^3^/s, the average leakage rate: 1.81 × 10^–5^ Pa·cm^3^/s. The fourth group of samples: 100 MPa/550 N, 350 °C under the bonding conditions of 1200 s, the maximum leakage rate measured: 1.83 × 10^–5^ Pa·cm^3^/s, the minimum leakage rate: 1.29 × 10^–5^ Pa·cm^3^/s, average leakage rate: 1.48 × 10^–5^ Pa·cm^3^/s. In conclusion, the leakage rate values measured by the four groups of different bonding parameters are all within the range of the leakage rate values required by the specification. It can be inferred from Figure 11 that with the increase in the bonding pressure, the average air tightness of the samples will gradually increase, and the average air tightness of the successfully bonded samples can meet the packaging requirements.

### 3.4. Failure Analysis

**Figure 12:**
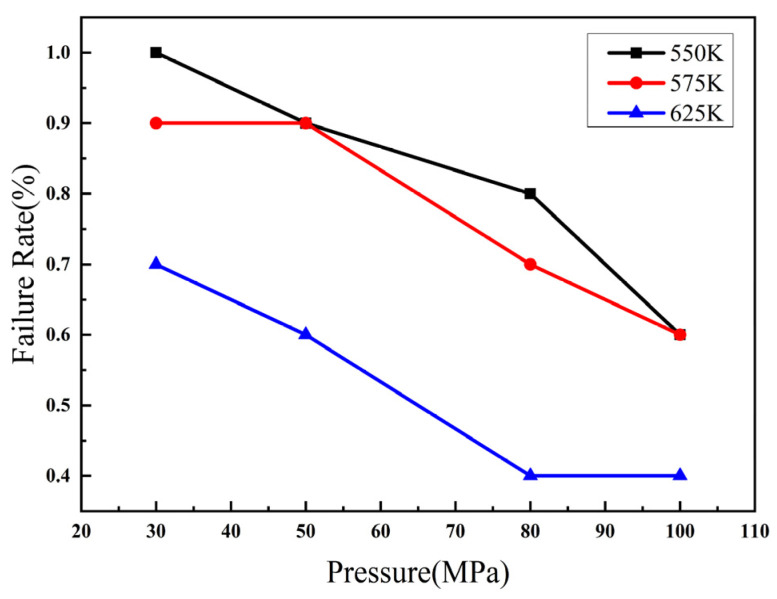
Influence of temperature and pressure on welding failure rate.

Owing to the high melting point and boiling point of Pt, the diffusion process is greatly restricted by temperature during the flip-chip welding process. Since the maximum welding temperature of flip-chip welding in the bulk silicon process does not exceed 400 °C, this work used a welding temperature of 250 °C–350 °C and a bonding pressure of 30 MPa–100 Mpa to explore the influence of different temperatures and pressures on welding failure rate. Bonding failure may be caused by a variety of reasons, and the failure may be manifested in that the top and bottom substrates do not adhere together or the tests such as shear force and air tightness cannot meet the test requirements. It was found that both the bonding pressure and the bonding temperature are positively correlated to the failure rate as shown in Figure 12. When the bonding temperature is 625 K and the bonding pressure is above 80 MPa, the failure rate is less than 0.4; under the same pressure conditions, when the temperature is less than 625 K, the failure rate is greater than 0.5. It shows that the failure rate is greatly affected by the bonding temperature, which should optimally be above 625 K. When the bonding pressure is less than 50 MPa, the failure rate of the bonding is more than 0.5, suggesting the optimal bonding pressure of Pt–Pt bonding is more than 50 MPa.

## 4. Conclusions

The Pt–Pt metal interconnection used flip-chip hot-press packaging technology to achieve a relatively stable interconnection at a temperature of 350 °C and pressure above 80 MPa. A series of experiments to evaluate its bonding performance was carried out. The thickness of the metal layer did not change significantly after bonding, and there was no Pt overflow at the bonding interface. In the case of bonding force of 550 N for 20 min, the shear resistance could reach up to 30 MPa. The shear experiment shows that the fractured interface is mostly on the silicon-metal layer, indicating that the interconnection interface after bonding is stable and does not easily fracture, indicating that the Pt–Pt metal bonding has a certain feasibility in packaging. The failure analysis experiment shows that the bonding pressure and temperature have an important influence on the failure rate, which is in line with the basic principle of metal welding. In addition, increasing the metal activity of the Pt metal interface may lower the requirements for bonding temperature and bonding pressure. Thus, these materials are considered worth exploring. We can plasma-treat the metal layer before bonding to improve the metal activity or find equipment that can increase the bonding temperature and bonding pressure to improve the quality of the bonding. In short, Pt–Pt metal bonding is a means of encapsulation that has great potential in the high-temperature environment in the future.

## Figures and Tables

**Table 1:** Case of bonding parameter.

Parameter	Force	Temperature	Time
Case1	165 N	350 °C	1200 s
Case2	275 N	350 °C	1200 s
Case3	440 N	350 °C	1200 s
Case4	550 N	350 °C	1200 s

**Table 2:** Case of leakage rate.

Parameter	Condition	Minimum Leakage Rate	Maximum Leakage Rate	Average Leakage Rate
Group 1	30 MPa/165 N	3.3 × 10^–4^	9.8 × 10^–4^	6.55 × 10^–4^
Group 2	50 MPa/275 N	3.3 × 10^–5^	5.9 × 10^–4^	3.115 × 10^–4^
Group 3	80 MPa/550 N	1.37 × 10^–5^	2.77 × 10^–5^	1.81 × 10^–5^
Group 4	100 MPa/550 N	1.29 × 10^–5^	1.83 × 10^–5^	1.48 × 10^–5^

## Data Availability

Not applicable.
